# Extracorporeal membrane oxygenation as acute rescue therapy for negative pressure pulmonary edema in the post anesthesia care unit: A case report

**DOI:** 10.1002/ccr3.7606

**Published:** 2023-06-30

**Authors:** Katrina J. Augustin, Christina M. Creel‐Bulos, Gaurav F. Budhrani, Casey F. Miller, Babar Fiza

**Affiliations:** ^1^ Department of Anesthesiology, Division of Critical Care Medicine Emory University Atlanta Georgia USA; ^2^ Department of Emergency Medicine Emory University Atlanta Georgia USA; ^3^ Department of Surgery, Division of Cardiothoracic Surgery Emory University Atlanta Georgia USA

**Keywords:** case report, extracorporeal life support, extracorporeal membrane oxygenation, negative pressure pulmonary edema

## Abstract

Negative pressure pulmonary edema (NPPE) may result in respiratory failure refractory to conventional management strategies. Venovenous extracorporeal membrane oxygenation (VV ECMO) can serve as a rescue therapy in cases of severe respiratory failure. Rapid initiation of VV ECMO can decrease morbidity and mortality while facilitating early liberation from mechanical ventilation and promoting early rehabilitation. We describe the successful utilization of VV ECMO as rescue therapy for severe NPPE‐induced hypoxic respiratory failure and peri‐arrest state in the postanesthesia care unit (PACU) in a patient with postextubation airway obstruction after undergoing patellar tendon repair.

## Graphical abstract

Negative pressure pulmonary edema induced acute respiratory failure with complete opacification of bilateral lung fields treated with venovenous extracorporeal membrane oxygenation.

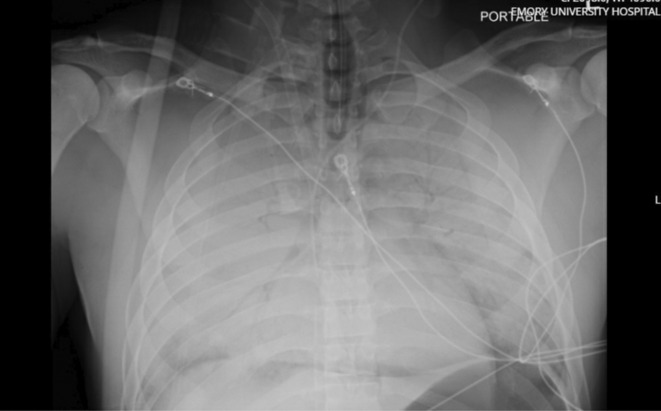

## 1 INTRODUCTION

Negative pressure pulmonary edema (NPPE), also known as post‐obstructive pulmonary edema, is a rare and life‐threatening cause of acute respiratory failure that is typically associated with upper airway obstruction (UAO), such as postextubation laryngospasm.^1^ The development of NPPE in the context of UOA involves a complex interplay of factors including dynamic shifts in intrathoracic pressure, a hyperadrenergic state, and elevated left ventricular (LV) transmural pressures.^1^ It is postulated that dynamic swings in intrathoracic pressure from deep negative inspiratory effort against an occluded airway or closed glottis facilitate fluid shifts with resultant increased venous return, elevated pulmonary venous pressures, and decreased perivascular interstitial hydrostatic pressure leading to the development of pulmonary edema.^1^ Furthermore, UAO induced hyperadrenergic state decreases venous capacitance, thus enhancing venous return and potentiating pulmonary congestion.^1^ Finally, elevated LV transmural pressure, from deep negative inspiratory pressure effort, as well as a hyperadrenergic state contributes to increased LV afterload. The increased LV afterload leads to decreased stroke volume, increased LV end diastolic pressure, and elevated left atrial pressure with resultant pulmonary edema.^1^

Conventional treatment of severe NPPE is focused on relieving the upper airway obstruction, positive pressure ventilation, and medication therapy including diuretics, with resolution of most cases. Case reports have described the need for delayed veno‐venous extracorporeal membrane oxygenation (VV ECMO) in cases of refractory hypoxia not responding to initial therapies.^2^, ^3^, ^4^, ^5^ However, the use of VV ECMO as acute rescue therapy in the post anesthesia care unit (PACU) for NPPE‐induced severe hypoxic respiratory failure, shock, and peri‐arrest physiology is yet to be described in the literature.

We describe the utilization of VV ECMO in the PACU as a rescue therapy for severe NPPE induced hypoxic respiratory failure and profound shock in a patient with post extubation airway obstruction after undergoing elective patellar tendon repair. Additionally, we discuss the challenges of systemic anticoagulation (SA) in the immediate postoperative period and early mechanical ventilation liberation facilitated by VV ECMO support.^6^

This manuscript adheres to the Consensus‐based Clinical Case Reporting Guidelines.^7^ Written Health Insurance Portability and Accountability Act authorization has been obtained for the publication of this case report.

## 2 CASE DESCRIPTION

A 29‐year‐old, American Society of Anesthesiology Physical Status Class II, man with a history of post‐traumatic stress disorder (PTSD) underwent elective open patellar tendon repair under general anesthesia at an outside hospital. The case was uneventful, and the patient was extubated before transfer to the PACU. On arrival to the PACU, he was noted to be apneic with concern for airway obstruction. Bag mask ventilation and narcan were administered. The patient developed worsening respiratory distress, profound hypoxemia (SpO_2_ < 85%), and copious pulmonary edema despite these interventions. Reintubation required several attempts due to the regurgitation of profuse pulmonary edema. The patient was given 120 mg of furosemide for diuresis. Multiple ventilation modes, including inverse ratio ventilation, were attempted without success, and hypoxemia persisted. Ventilator settings were maximized on a volume‐targeted pressure‐controlled mode with PEEP of 24 cm H_2_O and 100% FiO_2_ with a prolonged inspiratory time.

Despite maximal medical optimization, he had severe refractory hypoxia with subsequent circulatory shock. The patient required high dose vasopressor support including norepinephrine, epinephrine, and vasopressin infusions, along with bolus dosing of vasopressors to maintain adequate blood pressure. Given this, the ECMO team was consulted for emergent ECMO cannulation. The patient was deemed suitable for VV‐ECMO cannulation based on his Respiratory Survival Prediction Score (RESP) of three. RESP is a validated clinical scoring tool to predict in‐hospital survival after ECMO for acute respiratory failure. A RESP score of three is associated with a 76% survival rate and hence a favorable prognosis^8^ (Table 1). Given the hemodynamic instability, the decision was made to initiate VV ECMO therapy in the PACU. Based on the patient's BSA of 2.27 m^2^ (weight 102.3 kg, height 181.6 cm), normal hemoglobin, and presumed metabolic requirements, a 25 French multistage venous access cannula and a 22 French right internal jugular venous return cannula were chosen to optimize oxygen delivery. ECMO was initiated via a Cardiohelp system with the flow set to 5 L at 3460 RPMS and a sweep gas of 8 L. Before leaving the PACU, the Cardiohelp Heart‐Lung Support (HLS) circuit was noted to have a rapidly rising membrane pressure gradient (dP), from 20 mmHg to 80 mmHg, necessitating a circuit exchange due to concern for oxygenator thrombosis. The patient received 5000 units of heparin at the time of cannulation and an additional 5000 units followed by a heparin infusion at the time of oxygenator exchange. Subsequent dP maintained within the acceptable range of 20–30 mmHg.

**TABLE 1:** Patient's Respiratory Survival Prediction Score.

Variables	Patient specific variable	Points
Age	18–49 years old	0
Immunocompromise	No	0
Mechanical ventilation duration	<48 h	+3
Diagnosis	Other acute respiratory diagnosis	+1
History of central nervous system dysfunction	No	0
Acute associated nonpulmonary infection	No	0
Neuromuscular blockade before ECMO	Yes	+1
Nitric oxide before ECMO	No	0
Bicarbonate infusion before ECMO	No	0
Cardiac arrest before ECMO	No	0
PaCO_2_ ≥ 75 mmHg	Yes	−1
Peek inspiratory pressure ≥ 42 cm H20	Yes	−1
Total score (in hospital survival)	N/A	3 (76%)

**FIGURE 1:**
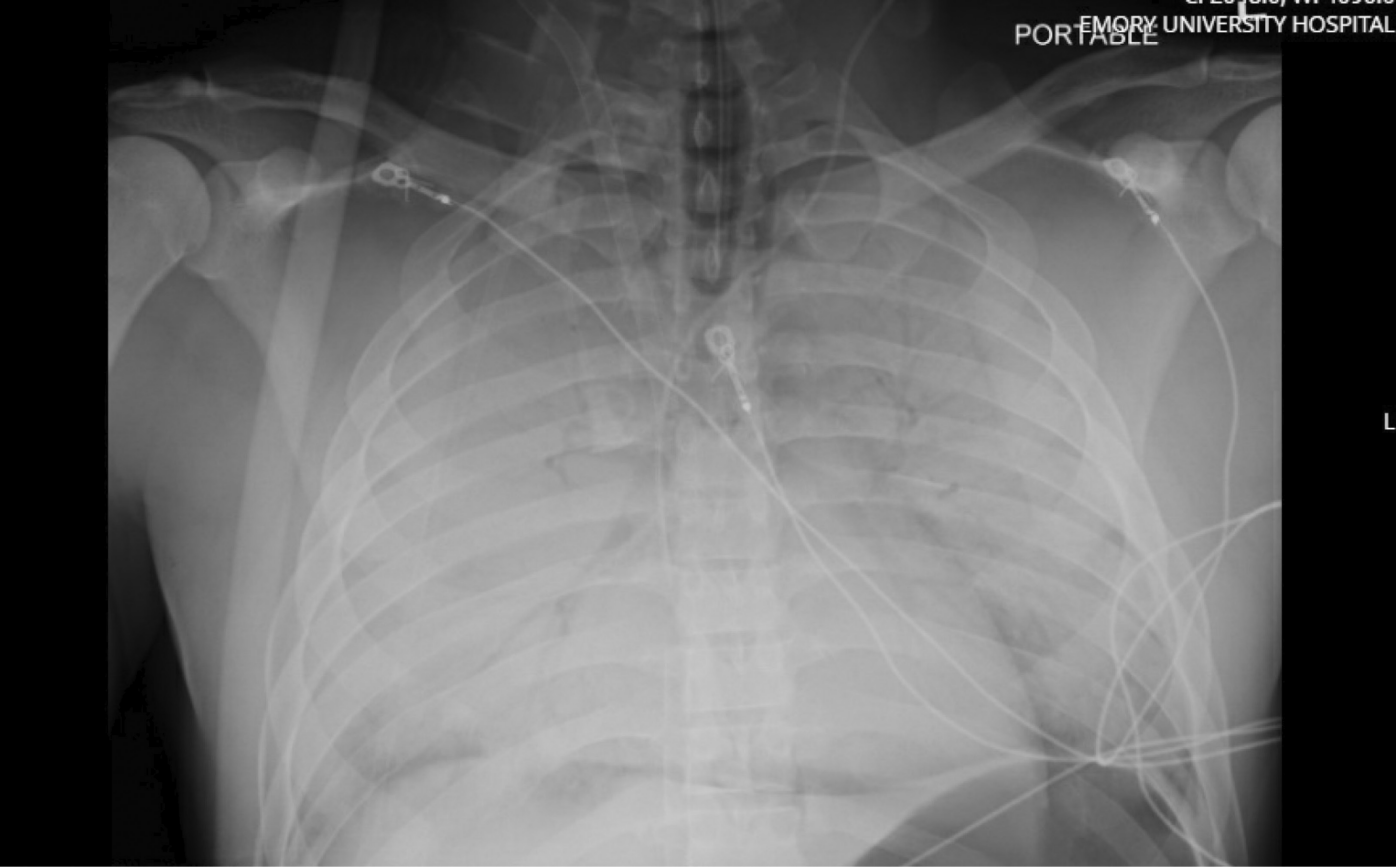
Portable chest radiograph from hospital day 1. This radiograph demonstrates complete opacification of bilateral lung fields with appropriately positioned ECMO cannulas 8.3 cm apart.

**FIGURE 2:**
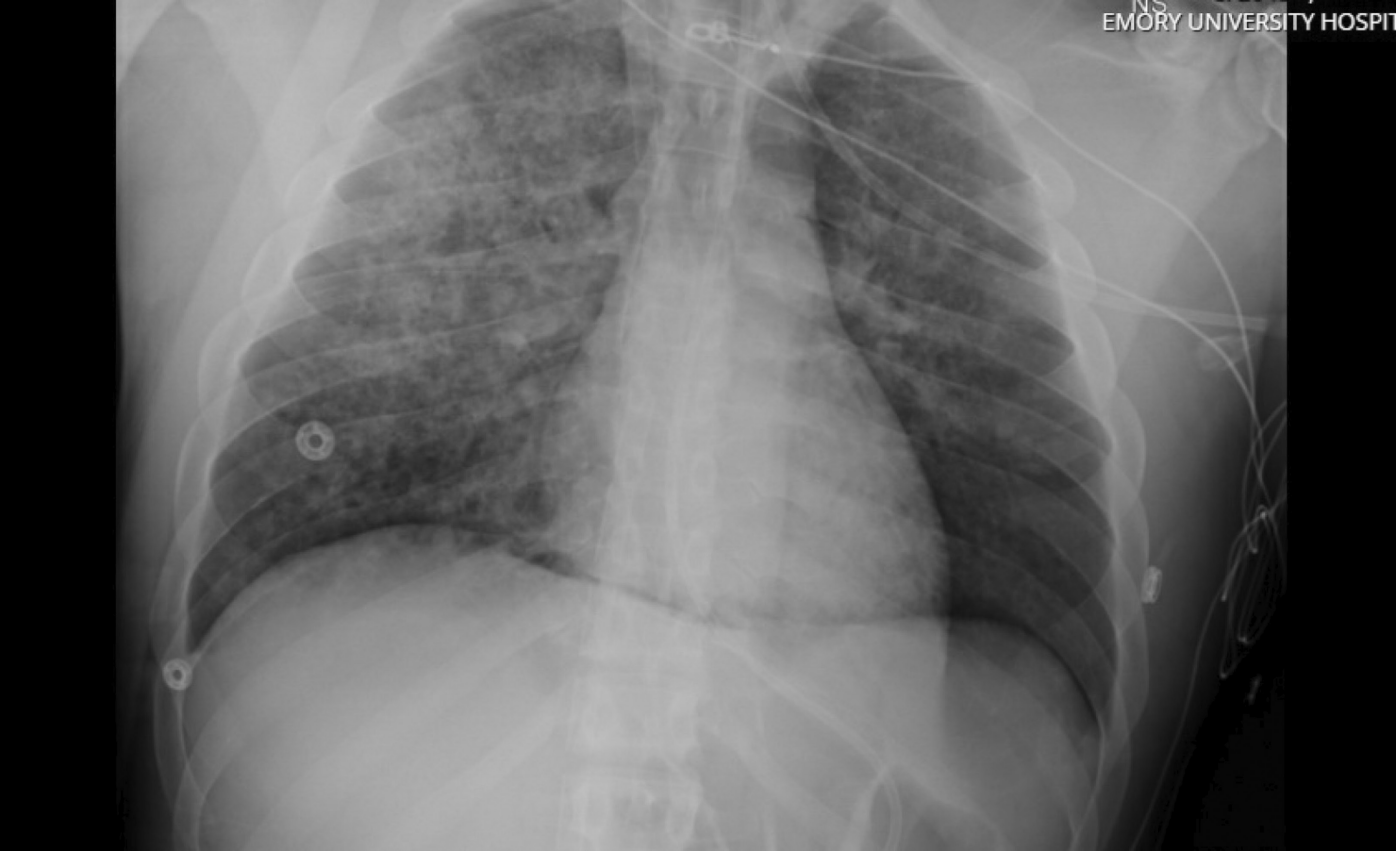
Portable chest radiograph hospital Day 3 (less than 48 h after cannulation). This radiograph demonstrates persistent extensive bilateral interstitial/airspace opacities with subsequent removal of endotracheal tube.

The patient was then transferred to the intensive care unit at our institution (Table 2), where he was transitioned to pressure control ventilation with PEEP of 15 cm H_2_O and inspiratory pressures of 16 cm H_2_O. Despite these settings, tidal volumes of only 20–70 mL could be achieved due to the poor pulmonary compliance. Initial chest radiograph (CXR) demonstrated complete opacification of bilateral lung fields (Figure 1). Within 24 h of cannulation, a rapid improvement in oxygenation was observed (Table 3). Vasopressor requirements were dramatically reduced, and he was weaned off all vasopressor support. Transthoracic echocardiogram revealed hyperdynamic left ventricular function, ejection fraction >70%, and normal right ventricular function. The acute kidney injury present on admission rapidly resolved, and he required diuresis for the first 48 h. Forty‐eight hours post cannulation, he was able to be extubated with ongoing support via VV ECMO despite his CXR demonstrating persistent extensive bilateral airspace opacities (Figure 2). Anticoagulation for the ECMO circuit was maintained with heparin infusion targeting aPTT goal of 40 s. A small amount of bleeding from the cannulation site occurred but resolved with suturing. No other hemorrhagic complications were experienced including at the operative site.

**TABLE 2:** Timeline of Relevant Clinical Events: Outline of key clinical events during the course of ICU admission and overall hospitalization.

Day	Events
1	V‐V ECMO cannulation (late afternoon)
	ECMO circuit exchange (approximately 30 min post cannulation)
	Transfer to our hospital/Admit to ICU
2	CXR with complete opacification of bilateral lung fields
	TTE with hyperdynamic EF >70%
	Transitioned to bilevel ventilation
	Vasopressors discontinued
3	Liberated from mechanical ventilation
	High flow nasal canula initiated
6	Decannulation from V‐V ECMO
	Transitioned to standard nasal canula at 1 L per minute
7	Transfer to floor
	No longer requiring oxygen therapy
8	Discharge to home

**TABLE 3:** Arterial Blood Gases Throughout Hospitalization.

Time	Fraction of inspired oxygen	ECMO flow (L)/Sweep gas (L) (100% FIO2 via sweep gas)	pH/pCO_2_/PaO_2_
Prior to cannulation	100%	0/0	(SpO_2_ remained <85%, ABG not available)
Immediate post cannulation	100%	4/4.5	7.22/64/154
<8 h post cannulation	70%	5/8	7.3/51/55
Day 3: post extubation	80%	5.25/3	7.44/44/188
Day 6: post decannulation	80%	0/0	7.49/39/141

**FIGURE 3:**
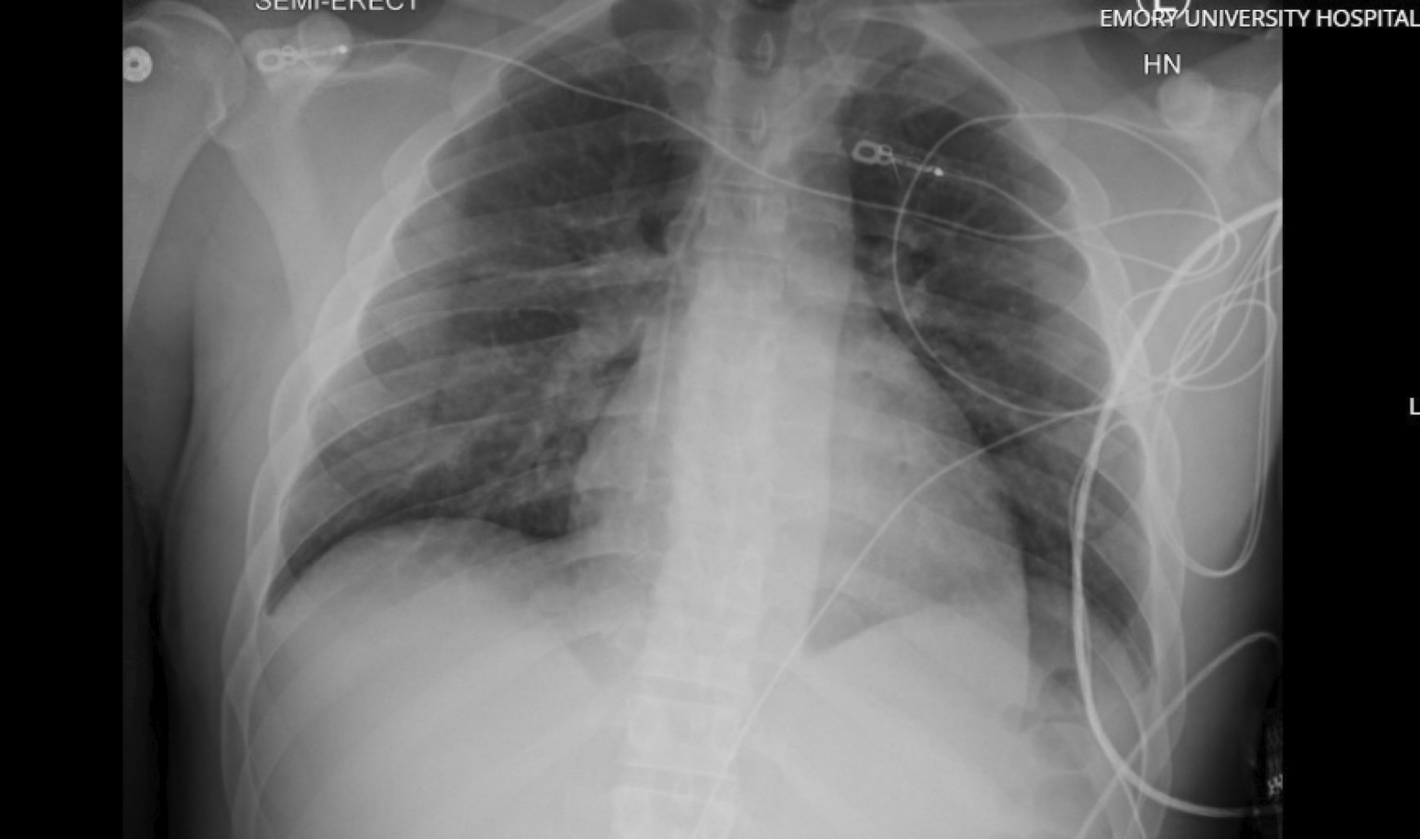
Portable chest radiograph from hospital Day 7 (day before discharge). This radiograph demonstrates interval improvement of pulmonary edema.

On Day 5, he tolerated a sweep trial and was subsequently liberated from ECMO. He briefly required oxygen via nasal cannula but was weaned to room air within the next 48 h with drastic improvements in his CXR (Figure 3). He was transferred to the floor on Day 7. On Day 8, he was discharged home‐neurologically intact without a supplemental oxygen requirement (Table 2).

## 3 DISCUSSION

This case highlights the potential benefits of early initiation of ECMO support in the perioperative period. The patient in this case was in extremis with severe hemodynamic instability and hypoxemia. In cases of severe hypoxemia, the inability to meet the high oxygen demand of the myocardium can result in hypotension and even cardiac arrest whether the cycle is not interrupted.^9^ The prompt initiation of VV ECMO in the PACU enabled the team to break this vicious cycle, preventing cardiac arrest and the potential for anoxic brain injury. Considering our patient's young age, absence of significant comorbidities, and normal or hyperactive cardiac function, the primary concern was severe hypoxia leading to impaired arterial oxygen content and subsequent hemodynamic instability. Recognizing this, we believed that addressing the underlying cause of circulatory shock and restoring tissue oxygenation would help resolve the shock state. Thus, VV ECMO rather than venoarterial ECMO therapy was selected as a treatment modality to augment oxygenation.

When initiating VV ECMO in the immediate postoperative period, the need for systemic anticoagulation (SA) poses a myriad of challenges. SA is generally required during ECMO therapies as blood exposed to foreign materials triggers the inflammatory response/coagulation cascade leading to prothrombotic state.^10^, ^11^ The risks of thromboembolic events such as circuit thrombosis, oxygenator failure, and venous thromboembolism must be weighed against the potential risks of major bleeding with SA in the postoperative period. With heparin bonded extracorporeal tubing and newer generation centrifugal pumps, there has been increased interest in minimizing SA in these patients and literature supports the feasibility of an anticoagulation free approach with no increased risk of thrombosis.^12^, ^13^ Unfortunately, our patient had a rapidly rising dP immediately post cannulation, raising concern for oxygenator thrombosis necessitating a circuit exchange. Given the concerns for a prothrombotic state, SA with heparin infusion was initiated and maintained until decannulation. The patient had no further significant oxygenator thrombosis with stable dPs and no post decannulation venous thromboembolism. Additionally, the patient had no hemorrhagic complications despite his recent orthopedic surgery.

Severe NPPE can significantly impair lung compliance, making achieving adequate gas exchange while avoiding further lung injury challenging. The classical approach to patients on VV ECMO has been to use “lung‐protective” or “ultra‐lung protective” ventilator settings in order to minimize ventilator‐induced lung injury. However, the optimal ventilation strategy for patients on ECMO is yet to be elucidated, with some centers utilizing “lung rest” while others focusing on “lung recruitment”.^14^ A recent study, assessing ventilation management in patients on VV ECMO with acute respiratory distress syndrome, found no significant association between ventilator settings during the first 2 days of ECMO support and survival.^14^ In this case, we opted for an individualized mechanical ventilation strategy to promote patient comfort, minimize patient‐ventilator desynchrony, and avoid any further lung injury.^15^ In patients with high respiratory drive, such as in this case, using low tidal volume ventilation could result in ventilator asynchrony, generation of high negative inspiratory pressures (NIPs), large swings in transpulmonary pressure, and worsening of underlying pulmonary edema.^15^

As illustrated by this case, utilization of VV ECMO can facilitate early extubation.^16^ Early liberation from mechanical ventilation and associated sedation can minimize the risk of delirium, critical illness myopathy/polyneuropathy, diaphragmatic dysfunction, and ventilator‐associated pneumonia.^17^ Early extubation also facilitates improved patient interaction, often an under‐appreciated benefit. Our patient was extubated within 36 h of the initial insult while being supported by VV ECMO. Early extubation, in this case, facilitated prompt cessation of sedation and early mobility, which minimized deconditioning in the setting of the patellar tendon repair. Additionally, the removal of sedation allowed the patient to interact with the medical team more effectively which mitigated patient's anxiety, a particularly crucial point in a patient with a history of PTSD.

In cases of severe NPPE with refractory hypoxia, despite conventional treatment with positive pressure ventilation, rapid initiation of VV ECMO in the postoperative period is not only feasible but can also decrease morbidity and mortality. VV ECMO facilitates early liberation from mechanical ventilation thus minimizing risk of delirium, critical illness myopathy/polyneuropathy, diaphragmatic dysfunction, and ventilator‐associated pneumonia while promoting early rehabilitation. Early initiation of ECMO therapy should be considered when presented with refractory respiratory failure.

## AUTHOR CONTRIBUTIONS

**Katrina J. Augustin:** Conceptualization; writing – original draft; writing – review and editing. **Christina M. Creel‐Bulos:** Conceptualization; writing – review and editing. **Gaurav F. Budhrani:** Conceptualization. **Casey F. Miller:** Conceptualization. **Babar Fiza:** Conceptualization; supervision; writing – review and editing.

## CONFLICT OF INTEREST STATEMENT

None.

## CONSENT

Written informed consent was obtained from the patient to publish this report in accordance with the journal's patient consent policy.

## Data Availability

All pertinent data available here and any additional data are available on request.
